# Alterations in the Gut Microbiota in Pregnant Women with Pregestational Type 2 Diabetes Mellitus

**DOI:** 10.1128/msystems.01146-22

**Published:** 2023-02-28

**Authors:** Yuan Ren, Lilan Hao, Juntao Liu, Pei Wang, Qiuxia Ding, Chen Chen, Yingna Song

**Affiliations:** a Department of Obstetrics and Gynecology, Peking Union Medical College Hospital, Chinese Academy of Medical Sciences & Peking Union Medical College, Beijing, China; b National Clinical Research Center for Obstetric & Gynecologic Diseases, Beijing, China; c BGI-Shenzhen, Shenzhen, China; Northern Arizona University

**Keywords:** bacterial metabolic pathway, gut microbiota, pregestational diabetes mellitus, pregnancy, taxonomic biomarker

## Abstract

Human gut dysbiosis is associated with type 2 diabetes mellitus (T2DM); however, the gut microbiome in pregnant women with pregestational type 2 diabetes mellitus (PGDM) remains unexplored. We investigated the alterations in the gut microbiota composition in pregnant women with or without PGDM. The gut microbiota was examined using 16S rRNA sequencing data of 234 maternal fecal samples that were collected during the first (T1), second (T2), and third (T3) trimesters. The PGDM group presented a reduction in the number of gut bacteria taxonomies as the pregnancies progressed. Linear discriminant analyses revealed that *Megamonas*, *Bacteroides*, and Roseburia intestinalis were enriched in the PGDM group, whereas Bacteroides vulgatus, Faecalibacterium prausnitzii, Eubacterium rectale, Bacteroides uniformis, Eubacterium eligens, *Subdoligranulum*, Bacteroides fragilis, *Dialister*, *Lachnospiraceae*, *Christensenellaceae* R-7, Roseburia inulinivorans, Streptococcus oralis, Prevotella melaninogenica, Neisseria perflava, Bacteroides ovatus, Bacteroides caccae, Veillonella dispar, and Haemophilus parainfluenzae were overrepresented in the control group. Correlation analyses showed that the PGDM-enriched taxa were correlated with higher blood glucose levels during pregnancy, whereas the taxonomic biomarkers of normoglycemic pregnancies exhibited negative correlations with glycemic traits. The microbial networks in the PGDM group comprised weaker microbial interactions than those in the control group. Our study reveals the distinct characteristics of the gut microbiota composition based on gestational ages between normoglycemic and PGDM pregnancies. Further longitudinal research involving women with T2DM at preconception stages and investigations using shotgun metagenomic sequencing should be performed to elucidate the relationships between specific bacterial functions and PGDM metabolic statuses during pregnancy and to identify potential therapeutic targets.

**IMPORTANCE** The incidence of pregestational type 2 diabetes mellitus (PGDM) is increasing, with high rates of serious adverse maternal and neonatal outcomes that are strongly correlated with hyperglycemia. Recent studies have shown that type 2 diabetes mellitus is associated with gut microbial dysbiosis; however, the gut microbiome composition and its associations with the metabolic features of patients with PGDM remain largely unknown. In this study, we investigated the changes in the gut microbiota composition in pregnant women with and without PGDM. We identified differential taxa that may be correlated with maternal metabolic statuses during pregnancy. Additionally, we observed that the number of taxonomic and microbial networks of gut bacteria were distinctly reduced in women with hyperglycemia as their pregnancies progressed. These results extend our understanding of the associations between the gut microbial composition, PGDM-related metabolic changes, and pregnancy outcomes.

## INTRODUCTION

Over the past several decades, the prevalence of type 2 diabetes mellitus (T2DM) has rapidly increased and become a worldwide public health issue (1). Similarly, the incidence of T2DM in pregnancy, or pregestational type 2 diabetes mellitus (PGDM), is increasing, and the rates of serious adverse maternal and neonatal outcomes remain high (2, 3). PGDM is associated with several pregnancy complications, including pre-eclampsia, preterm delivery, and congenital malformations (4–6). Approximately half of the pregnancies affected by PGDM are complicated with macrosomia, which increases not only the risk of birth trauma and cesarian sections among mothers but also the risk of metabolic syndrome and cardiovascular disease in their offspring (7). The high prevalence of these adverse outcomes is strongly correlated with poor glycemic control (8); therefore, strict glycemic monitoring and management are critical for preventing these pregnancy-related complications.

The gut microbiota is considered essential for modulating host metabolism. With the emergence of 16S rRNA sequencing and shotgun metagenomic sequencing, the composition, functions, and role of gut microbiota in health and metabolic disorders have been intensively investigated. Recent studies have further identified the gut microbiome as a potential driving factor in the pathogenesis of T2DM (9). T2DM is associated with gut microbial dysbiosis, including reduced bacterial diversity, decreased *Firmicutes*/*Bacteroidetes* ratios, enriched opportunistic pathogens, reduced abundance of butyrate-producing bacteria, and altered expression levels of microbial genes and metabolic pathways involved in low-grade inflammation (10–14).

Several studies have reported a profound remodeling of the gut microbiota, such as increases in β-diversity and decreases in α-diversity, during pregnancy. The abundance of butyrate-producing bacteria is also reduced and is similar to that in nonpregnant adults with metabolic syndrome. These changes are beneficial to healthy pregnancies because they increase nutrient absorption and support fetal growth (15–19).

PGDM involves more serious manifestations and requires more complicated clinical management than gestational diabetes mellitus as a transient metabolic disorder (20). However, studies have yet to elucidate the composition of the gut microbiome or its associations with the metabolic features of pregnant women with PGDM.

In this study, we performed 16S rRNA amplicon sequencing on fecal samples collected from 80 pregnant Chinese women with (*n* = 20) or without (*n* = 60) PGDM in the first (T1), second (T2), and third (T3) trimesters of pregnancy. We aimed to reveal the composition and dynamics of the gut microbiota in PGDM and healthy pregnancies and determine the variations in the gut microbiota based on the maternal metabolic status. Our findings provide insights into the gut microbial transformations during pregnancy and their associations with the clinical outcomes of PGDM.

## RESULTS

### Clinical characteristics of the participants.

**FIG 1:**
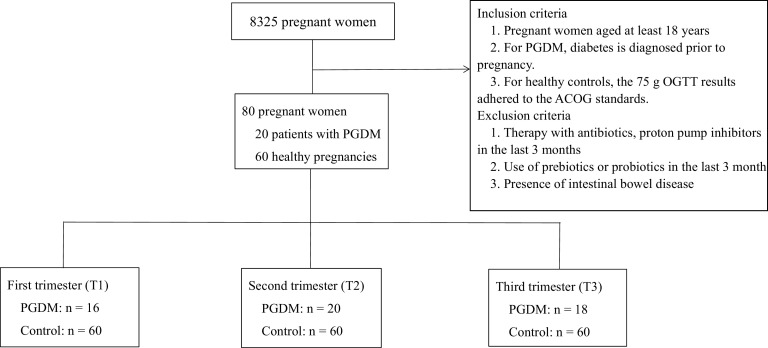
Participants and sample statistics. PGDM, pregestational diabetes mellitus; 75-g OGTT, 75-g oral glucose tolerance test; ACOG, American College of Obstetricians and Gynecologists.

Figure 1 describes the participants enrolled in this study, including 20 women with PGDM and 60 women with healthy pregnancies. The women in the PGDM group had a median T2DM duration of 24 months and were more likely to be diagnosed with polycystic ovary syndrome than the women in the healthy control group. Approximately 75% of the women with PGDM used insulin alone to control their blood glucose levels, while 20% of the women with PGDM used insulin combined with metformin to achieve better glycemic control. The two groups were similar in age, gravidity, and parity (Table 1). The preconception weights, body mass indices, and systolic and diastolic blood pressures were higher in the PGDM group than in the healthy control group. These characteristics, along with the high prevalence of polycystic ovary syndrome in the PGDM group, are all high-risk factors for T2DM and cannot be balanced. The weight gains during pregnancy were comparable between the two groups, which indicated ideal glycemic control in the women with PGDM. Consistent with those reported in clinical practice, the times of delivery were earlier in the PGDM group than in the healthy controls, while the neonatal birth weights and numbers of admissions to neonatal intensive care units (NICUs) were similar between the two groups. The PGDM cases had higher levels of fasting blood glucose (FBG) and glycated albumin (GA) throughout the pregnancies than the control group, but the levels remained within the range that is considered acceptable in the PGDM guidelines of the American College of Obstetricians and Gynecologists. Moreover, the liver and renal functions and C-reactive protein levels did not differ between the groups. These findings indicate that the hyperglycemic pregnancies included in our study may be defined as cases with managed PGDM.

**TABLE 1:** Clinical characteristics of the study population^*a*^

Characteristic	PGDM (*n* = 20)	Control (*n* = 60)	*P*
Baseline characteristics			
Age (yr)	34.5 (30.0–36.0)	31.0 (29.3–34.8)	0.073
Gravidity	1.0 (1.0–2.0)	1.0 (1.0–2.0)	0.095
Parity	1.0 (1.0–1.8)	1.0 (1.0–1.0)	0.199
Duration of T2DM (month)	24.0 (5.3–48.0)	NA	NA
No. (%) with PCOS	5 (25.0)	4 (6.7)	0.039
No. (%) with family history of diabetes	6 (30.0)	8 (13.3)	0.174
Anthropometrics and perinatal outcomes			
Ht (cm)	162.2 ± 6.3	163.8 ± 5.2	0.239
Wt (kg)	78.2 ± 12.0	71.2 ± 8.8	0.006
Preconception wt (kg)	64.0 ± 9.8	58.5 ± 7.0	0.007
Preconception BMI (kg/m^2^)	24.2 (22.1–25.5)	21.7 (19.6–23.2)	0.001
Wt gain (kg)	14.0 (11.3–15.4)	12.3 (10.0–15.5)	0.266
SBP (mm Hg)	119.0 (112.0–141.0)	113.0 (106.5–120.8)	0.022
DBP (mm Hg)	81.0 (75.3–90.5)	70.5 (65.0–77.0)	<0.001
No. (%) with IVF-ET	2 (10.0)	5 (8.3)	1.000
No. (%) with preconception glycemic control			
No medication	5 (25.0)	NA	
Insulin	6 (30.0)	NA	
Metformin	5 (25.0)	NA	
Insulin and metformin	4 (20.0)	NA	
No. (%) with glycemic control in pregnancy			
No medication	1 (5.0)	NA	
Insulin aspart	1 (5.0)	NA	
Insulin detemir	1 (5.0)	NA	
Insulin aspart and insulin detemir	13 (65.0)	NA	
Insulin and metformin	4 (20.0)	NA	
Lactulose administration	2 (10.0)	4 (6.7)	1.000
Gestational wks of delivery	38.4 (37.6–39.3)	39.5 (38.9–40.4)	0.001
No. (%) with mode of delivery			0.104
Vaginal delivery	10 (50.0)	42 (70.0)	
Caesarean section	10 (50.0)	18 (30.0)	
Neonatal birth wt (g)	3,375.0 (2,915.0–3,557.5)	3,285.0 (3,032.5–3,600.0)	0.807
No. (%) with NICU admission	4 (20.0)	5 (8.3)	0.307
Biochemistry characteristics			
Glucose at 0 h (mmol/L)	8.1 (5.5–9.8)	4.7 (4.3–5.3)	<0.001
Glucose at 2 h (mmol/L)	11.1 ± 4.0	6.5 ± 0.7	<0.001
Insulin at 0 h (mIU/mL)	10.1 (7.9–13.6)	NA	NA
Insulin at 2 h (mIU/mL)	57.1 (36.4–75.7)	NA	NA
FBG in first trimester (mmol/L)	5.8 (5.4–6.4)	4.7 (4.3–4.9)	<0.001
GA in first trimester (mmol/L)	15.2 (14.6–16.2)	13.4 (12.8–14.2)	<0.001
HbA1c in first trimester (%)	6.0 (5.6–6.2)	NA	NA
FBG in second trimester (mmol/L)	5.7 (5.1–6.0)	4.5 (4.1–4.7)	<0.001
GA in second trimester (mmol/L)	14.3 (13.5–15.2)	13.2 (12.3–13.6)	<0.001
FBG in third trimester (mmol/L)	5.7 (4.9–6.4)	4.7 (4.2–5.0)	<0.001
GA in third trimester (mmol/L)	13.4 ± 2.5	12.7 ± 1.0	0.014
ALT (U/L)	15.5 (11.0–18.5)	14.0 (10.3–19.0)	0.709
ALB (g/L)	44.0 (42.1–44.8)	43.5 (42.0–45.0)	0.707
Serum creatinine (μmol/L)	50.6 ± 9.4	50.8 ± 6.3	0.873
TC (mmol/L)	4.8 ± 1.0	5.2 ± 1.4	0.262
TG (mmol/L)	1.8 (0.8–2.4)	1.5 (0.8–1.9)	0.773
HDL (mmol/L)	1.3 (1.0–1.7)	1.8 (1.4–2.0)	0.003
LDL (mmol/L)	2.6 ± 0.7	2.7 ± 0.9	0.805
Apolipoprotein A1 (g/L)	1.3 (1.2–1.9)	1.9 (1.6–2.5)	0.001
Apolipoprotein B (g/L)	0.9 ± 0.2	0.9 ± 0.2	0.667
Lipoprotein-a (mg/L)	80.0 (28.5–136.7)	161.9 (38.8–236.1)	0.224
CRP (mg/L)	2.1 (1.4–2.7)	2.2 (0.5–5.0)	0.996
FFA (μmol/L)	660.1 ± 221.1	316.7 ± 160.1	<0.001

a Normally distributed continuous variables are reported as mean ± standard deviation and were analyzed using Student’s *t* test; other values are reported as medians with interquartile ranges and were analyzed using the Mann-Whitney U test. Qualitative data are presented as number (percent) and were analyzed by χ^2^ test or Fisher’s exact-probability test. PGDM, pregestational diabetes mellitus; T2DM, type 2 diabetes mellitus; NA, not available; PCOS, polycystic ovarian syndrome; BMI, body mass index; SBP, systolic blood pressure; DBP, diastolic blood pressure; IVF-ET, *in vitro* fertilization and embryo transplantation; NICU, neonatal intensive care unit; FBG, fasting blood glucose; GA, glycated albumin; HbA1c, glycated hemoglobin; ALT, alanine aminotransferase; ALB, serum albumin; TC, total cholesterol; TG, triglycerides; HDL, high-density cholesterol; LDL, low-density cholesterol; CRP, C-reactive protein; FFA, free fatty acid.

### Altered gut microbiota composition in women with PGDM.

**FIG 2:**
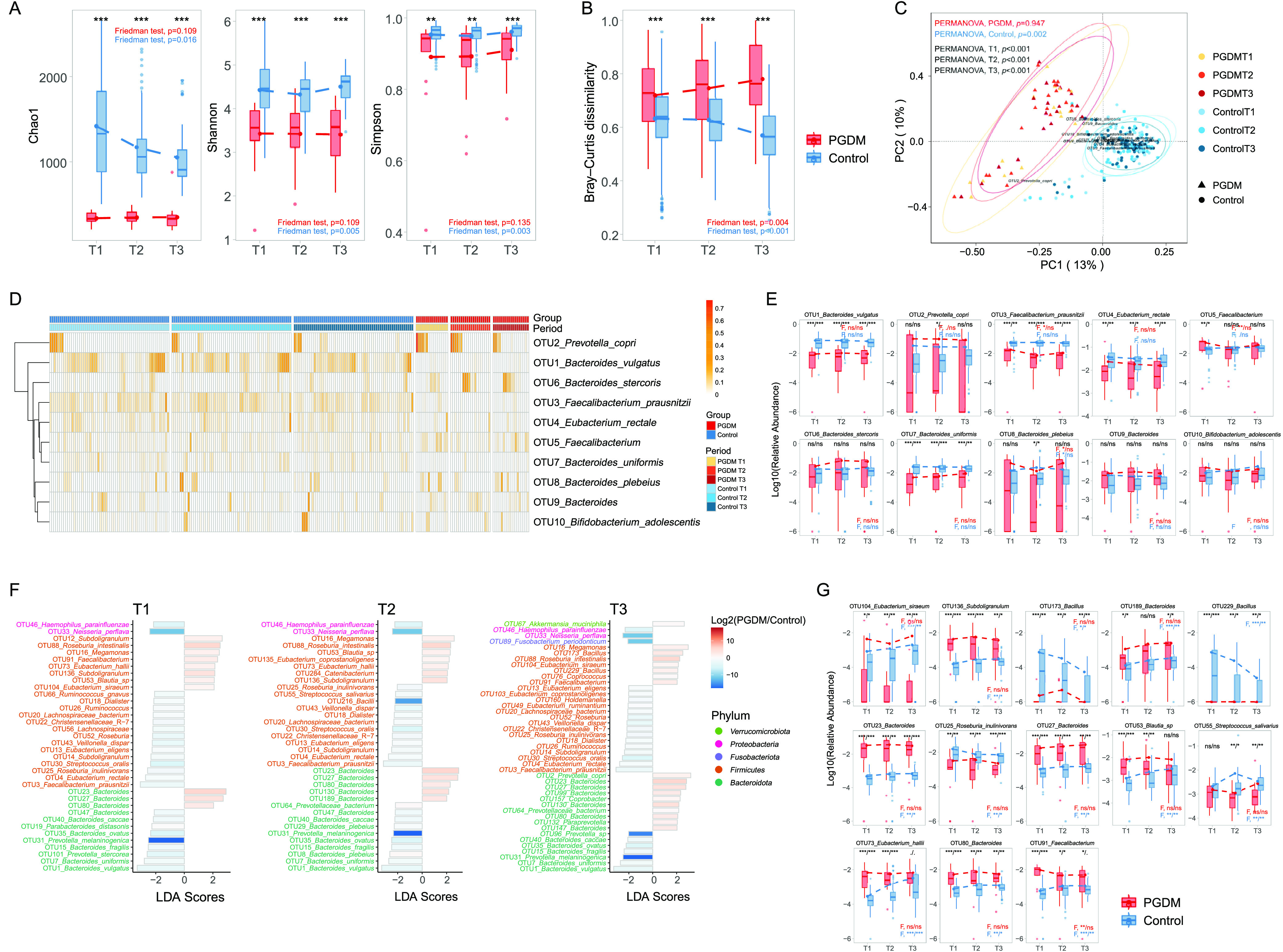
Gut microbiota composition in pregnant women with or without PGDM. (A) Comparison of alpha diversity indices (Chao1, Shannon, and Simpson) within and between the PGDM (red) and control (blue) groups from T1 to T3. (B) Bacterial community dissimilarities within and between the PGDM (red) and control (blue) groups from T1 to T3. (C) PCoA of the Bray-Curtis dissimilarities of the two groups from T1 to T3; ellipses represent 95% confidence intervals (CIs). (D) Heat map of the top 10 OTUs in both groups in different trimesters. (E) Comparison of the relative abundances of the top 10 OTUs within and between the PGDM (red) and control (blue) groups from T1 to T3. (F) Linear discriminant analysis score of the taxonomic biomarkers for the women with PGDM and healthy controls. The colors of the bars indicate the log_2_ fold changes in the relative abundances for the taxonomic biomarkers between the women with and without PGDM. The color of the *y* axis label indicates the phylum of each OTU. (G) Comparison of the relative abundances of the taxonomic biomarkers within and between the PGDM (red) and control (blue) groups from T1 to T3. The dotted line of the graph with each box plot represents the average. F, Friedman test. Significances based on *P* and *Q* values are separated by a slash. ns, not significant (*P* or *Q* > 0.1); *, *P* or *Q *< 0.05; **, *P* or *Q *< 0.01; ***, *P* or *Q *< 0.001.

A total of 13,252,913 high-quality reads were detected in 234 fecal samples collected from all of the participants from T1 to T3, with an average yield of 56,636 reads per sample. The samples were checked for quality and clustered into 9,454 operational taxonomic units (OTUs) (see Table S1 in the supplemental material). Alpha diversity analysis showed that the women with PGDM had lower richness (lower Chao1 index, *P < *0.001 in all trimesters) and diversity (lower Shannon index, *P < *0.001 in all trimesters; lower Simpson index, *P = *0.001 in T1, *P = *0.004 in T2, and *P < *0.001 in T3) during pregnancy than the healthy controls (Fig. 2A). A permutational multivariate analysis of variance (PERMANOVA) detected marked dissimilarities in the gut microbial composition between the two groups in different trimesters (*P < *0.001 in all trimesters) (Fig. 2B). Principal-coordinate analysis (PCoA) plots further showed group-specific clustering in the samples from women with or without PGDM (*P < *0.001 in all trimesters) (Fig. 2C).

10.1128/msystems.01146-22.3 TABLE S1 OTU statistics. Download Table S1, XLSX file, 0.02 MB. Copyright © 2023 Ren et al. 2023 Ren et al. https://creativecommons.org/licenses/by/4.0/ This content is distributed under the terms of the Creative Commons Attribution 4.0 International license.

Comparison of the compositions of the top 10 bacterial phyla in the two groups revealed that the gut microbiota mainly comprised *Bacteroidetes*, *Firmicutes*, *Proteobacteria*, and *Actinobacteria* at the phylum level (Fig. S1A; Table S2). In the PGDM group, the abundance of *Bacteroidetes* increased between trimesters (Fig. S1B). The *Firmicutes*/*Bacteroidetes* ratio significantly decreased in T2 (*P = *0.0037) and T3 (*P = *0.0065) compared with that in the healthy controls (Fig. S1C). We further identified the top 10 OTUs in the pregnant women with or without PGDM. OTU1 (Bacteroides vulgatus) and OTU2 (Prevotella copri) were the most prevalent OTUs, followed by OTU3 (Faecalibacterium prausnitzii), OTU4 (Eubacterium rectale), OTU5 (*Faecalibacterium*), OTU6 (Bacteroides stercoris), OTU7 (Bacteroides uniformis), OTU8 (Bacteroides plebeius), OTU9 (*Bacteroides*), and OTU10 (Bifidobacterium adolescentis) (Fig. 2D). Most of these OTUs were comparable between the two groups. Conversely, the relative abundances of OTU1 (Bacteroides vulgatus), OTU3 (Faecalibacterium prausnitzii), OTU4 (Eubacterium rectale), and OTU7 (Bacteroides uniformis) were lower in the patients with hyperglycemic pregnancies than in the healthy controls (Fig. 2E; Table S3).

10.1128/msystems.01146-22.1 FIG S1 Top 10 phyla of the gut microbiota in pregnant women with or without PGDM. (A) Heat map of the top 10 phyla in both groups in different trimesters. (B) Comparison of the relative abundances of the top 10 phyla within and between the PGDM (red) and control (blue) groups from T1 to T3. (C) Comparison of the *Firmicutes*/*Bacteroidetes* ratio between the PGDM (red) and control (blue) groups from T1 to T3. (D) Number of stage-specific OTUs in the two groups at the significance thresholds *P* < 0.05 and *Q* < 0.05. Red and blue represent PGDM and healthy controls, respectively. The dotted line of the graph with each box plot represents the average. F, Friedman test. Significances of *P* values and *Q* values are separated by a slash. ns, not significant (*P* or *Q* > 0.1); *, *P* or *Q *< 0.05; **, *P* or *Q *< 0.01; ***, *P* or *Q *< 0.001. Download FIG S1, EPS file, 1.1 MB. Copyright © 2023 Ren et al. 2023 Ren et al. https://creativecommons.org/licenses/by/4.0/ This content is distributed under the terms of the Creative Commons Attribution 4.0 International license.

10.1128/msystems.01146-22.4 TABLE S2 Comparison of the relative abundances of the predominant phyla within and between the PGDM and control groups from T1 to T3. Download Table S2, XLSX file, 0.01 MB. Copyright © 2023 Ren et al. 2023 Ren et al. https://creativecommons.org/licenses/by/4.0/ This content is distributed under the terms of the Creative Commons Attribution 4.0 International license.

10.1128/msystems.01146-22.5 TABLE S3 Comparison of the relative abundances of the taxonomic biomarkers within and between the PGDM and control groups from T1 to T3. Download Table S3, XLSX file, 0.1 MB. Copyright © 2023 Ren et al. 2023 Ren et al. https://creativecommons.org/licenses/by/4.0/ This content is distributed under the terms of the Creative Commons Attribution 4.0 International license.

Linear discriminant analysis revealed multiple taxonomic biomarkers in the two groups in different trimesters (Fig. 2F; Table S4). In T1, OTU12 (*Subdoligranulum*), OTU16 (*Megamonas*), OTU23 (*Bacteroides*), OTU27 (*Bacteroides*), OTU53 (*Blautia* sp.), OTU73 (Eubacterium hallii), OTU80 (*Bacteroides*), OTU88 (Roseburia intestinalis), OTU91 (*Faecalibacterium*), OTU104 (Eubacterium siraeum), and OTU136 (*Subdoligranulum*) were more abundant in the PGDM group than in the healthy controls. Among these OTUs, OTU16 (*Megamonas*), OTU23 (*Bacteroides*), OTU27 (*Bacteroides*), OTU80 (*Bacteroides*), and OTU88 (Roseburia intestinalis) were consistently enriched in patients with hyperglycemia from T1 to T3. Conversely, 25 biomarkers had a high relative abundance in the healthy controls in T1. Among them, OTU1 (Bacteroides vulgatus), OTU3 (Faecalibacterium prausnitzii), OTU4 (Eubacterium rectale), OTU7 (Bacteroides uniformis), OTU13 (Eubacterium eligens), OTU14 (*Subdoligranulum*), OTU15 (Bacteroides fragilis), OTU18 (*Dialister*), OTU20 (*Lachnospiraceae*), OTU22 (*Christensenellaceae* R-7), OTU25 (Roseburia inulinivorans), OTU30 (Streptococcus oralis), OTU31 (Prevotella melaninogenica), OTU33 (Neisseria perflava), OTU35 (Bacteroides ovatus), OTU40 (Bacteroides caccae), OTU43 (Veillonella dispar), and OTU46 (Haemophilus parainfluenzae) were consistently enriched in the women with normal glucose levels as their pregnancies progressed from T1 to T3.

10.1128/msystems.01146-22.6 TABLE S4 Linear discriminant analysis score of the taxonomic biomarkers for the women with PGDM and healthy controls. Download Table S4, XLSX file, 0.02 MB. Copyright © 2023 Ren et al. 2023 Ren et al. https://creativecommons.org/licenses/by/4.0/ This content is distributed under the terms of the Creative Commons Attribution 4.0 International license.

We further used LASSO (least absolute shrinkage and selection operator) regression analysis in identifying group-specific taxa to exclude the potential bias introduced by the unbalanced body mass index (BMI) and polycystic ovarian syndrome (PCOS) prevalence between the two groups (Fig. S2A; Table S5). In T1, OTU2 (Prevotella copri), OTU23 (*Bacteroides*), OTU32 (*Prevotella*), OTU91 (*Faecalibacterium*), OTU130 (*Bacteroides*), OTU228 (*Blautia*), OTU482 (*Lachnoclostridium*), OTU702 (*Lachnoclostridium*), and OTU711 (*Ruminococcus*) were more abundant in the PGDM group than in the healthy controls. Among these OTUs, OTU23 (*Bacteroides*), OTU32 (*Prevotella*), and OTU482 (*Lachnoclostridium*) were consistently enriched in patients with hyperglycemia, independent of trimester. Conversely, OTU112 (*Lachnoclostridium*) and OTU240 (Catonella morbi) had a high relative abundance in the healthy controls in T1. We did not find any OTUs that were consistently enriched in women with normal glucose levels as their pregnancies progressed from T1 to T3. Specifically, in the control group, OTU3 (Faecalibacterium prausnitzii), OTU240 (Catonella morbi), and OTU429 (*Blautia*) were enriched in T2, while OTU1 (Bacteroides vulgatus), OTU3 (Faecalibacterium prausnitzii), OTU30 (Streptococcus oralis), OTU118 (*Porphyromonas*), and OTU429 (*Blautia*) were enriched in T3.

10.1128/msystems.01146-22.2 FIG S2 LASSO regression analysis of the taxonomic biomarkers for the women with or without PGDM. (A) LASSO regression coefficients of the taxonomic biomarkers for the women with PGDM and healthy controls. The colors of the bars indicate the log_2_ fold changes in the relative abundances for the taxonomic biomarkers between the women with and without PGDM. The color of the *y* axis label indicates the phylum of each OTU. (B) Comparison of the relative abundances of the taxonomic biomarkers identified by LASSO regression coefficients within and between the PGDM (red) and control (blue) groups from T1 to T3. The dotted line of the graph with each box plot represents the average. F, Friedman test. Significances of *P* and *Q* values are separated by a slash. ns, not significant (*P* or *Q* > 0.1); *, *P* or *Q *< 0.05; **, *P* or *Q *< 0.01; ***, *P* or *Q *< 0.001. Download FIG S2, EPS file, 0.4 MB. Copyright © 2023 Ren et al. 2023 Ren et al. https://creativecommons.org/licenses/by/4.0/ This content is distributed under the terms of the Creative Commons Attribution 4.0 International license.

10.1128/msystems.01146-22.7 TABLE S5 LASSO regression coefficients of the taxonomic biomarkers for the women with PGDM and healthy controls. Download Table S5, XLSX file, 0.01 MB. Copyright © 2023 Ren et al. 2023 Ren et al. https://creativecommons.org/licenses/by/4.0/ This content is distributed under the terms of the Creative Commons Attribution 4.0 International license.

### Dynamics of the gut microbiota composition from T1 to T3.

Our analysis of the intertrimester dynamics of the gut microbiota from T1 to T3 in both groups revealed no significant differences in the alpha diversity in the PGDM group in different trimesters. Conversely, the Chao1 index significantly decreased (*P = *0.016, Friedman test) and the Shannon and Simpson indices increased (*P = *0.005; *P = *0.003, Friedman test) (Fig. 2A) from T1 to T3 in the healthy controls. Furthermore, the dissimilarities in the gut microbiome during pregnancy showed an increase with gestational age in women with PGDM (PERMANOVA, *P = *0.004). Conversely, it distinctly decreased in the healthy controls from T1 to T3 (PERMANOVA, *P < *0.001) (Fig. 2B).

At the phylum level, the top 10 phyla in the women with PGDM in different trimesters were not significantly different. *Verrucomicrobiota*, however, was less abundant in T3 than in T1 (*P = *0.003). In the control group, *Acidobacteria* (*P = *0.001), *Gemmatimonadetes* (*P = *0.001), and *Verrucomicrobia* (*P = *0.008) were less abundant in T3 than in T1 (Fig. S1B; Table S2). In the PGDM and control groups, the shift in the *Firmicutes*/*Bacteroidetes* ratio from T1 to T3 was comparable (*P = *0.223 in the PGDM group and *P = *0.296 in the control group) (Fig. S1C).

A longitudinal investigation into the changes in the OTUs in both groups as the pregnancies progressed revealed that 25 OTUs in the PGDM group presented different relative abundances with gestational ages and at a nominal significance threshold. None of these OTUs withstood the correction for multiple testing. In the control group, 218 stage-specific OTUs were found, and 157 OTUs remained after multiple testing corrections (Fig. S1D), 13 of which were also identified as taxonomic biomarkers between the two groups (Fig. 2G; Table S3). In the women with normoglycemia, the relative abundances of nine PGDM-enriched taxa, namely, OTU23 (*Bacteroides*), OTU27 (*Bacteroides*), OTU53 (*Blautia* sp.), OTU73 (Eubacterium hallii), OTU80 (*Bacteroides*), OTU91 (*Faecalibacterium*), OTU104 (Eubacterium siraeum), OTU136 (*Subdoligranulum*), and OTU189 (*Bacteroides*), increased from T1 to T3, whereas the relative abundance of one taxon in the healthy group, namely, OTU25 (Roseburia inulinivorans), decreased from T1 to T3. Among these taxa, three OTUs, namely, OTU23 (*Bacteroides*), OTU27 (*Bacteroides*), and OTU91 (*Faecalibacterium*), withstood the correction of the LASSO regression analysis (Fig. S2B; Tables S2 and S5). These taxonomic changes resembled those in the gut microbiota composition in the PGDM group.

### Associations between the gut microbial composition and glycemic traits.

**FIG 3:**
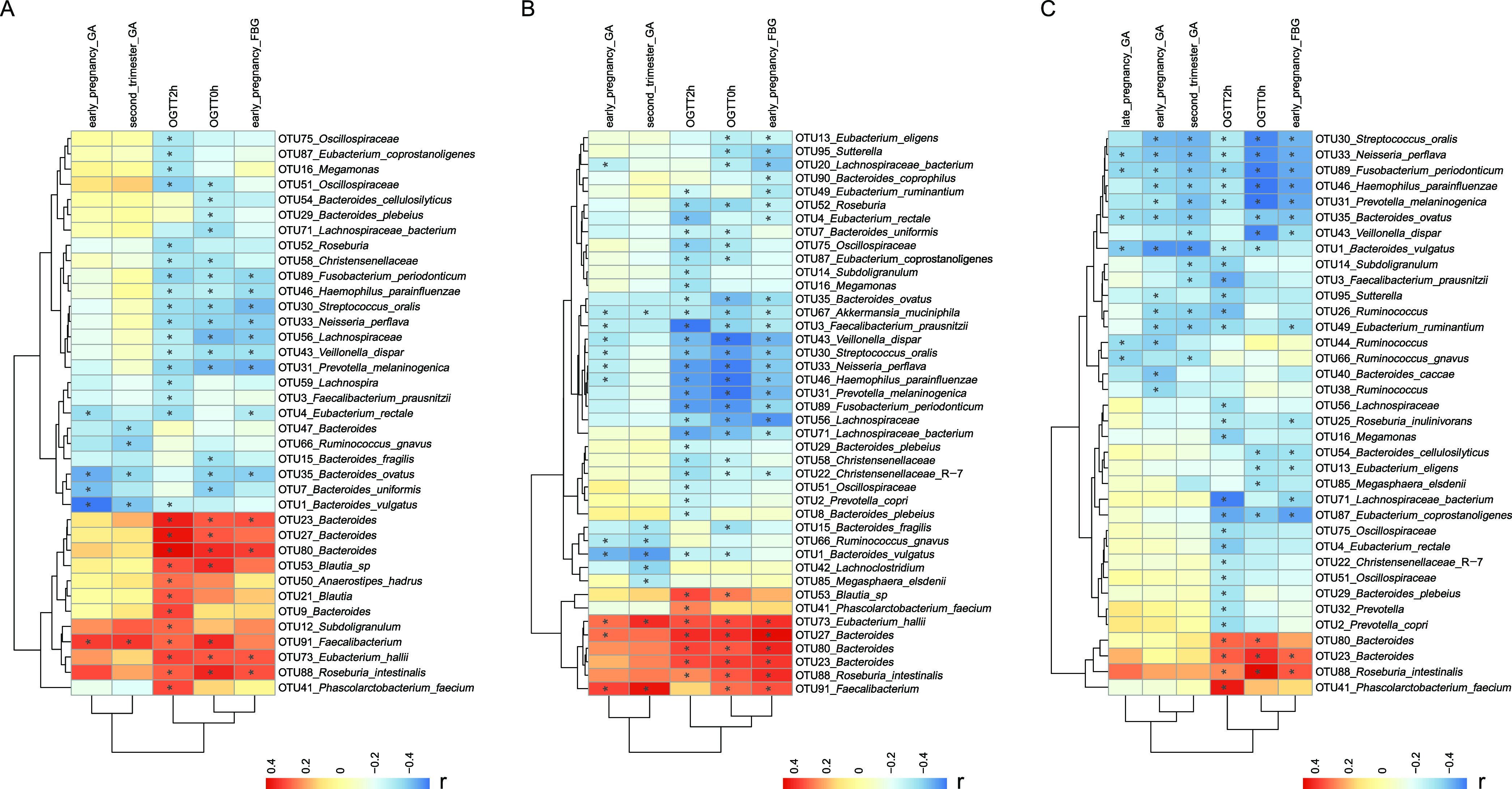
Spearman’s rank correlation heat map of specific OTUs and clinical indices regardless of PGDM status in the first (A), second (B), and third (C) trimesters. Features that have at least one significant sign are shown. The colors of the cells represent the *r* value of the correlation coefficient. *, *P < *0.05.

Spearman’s correlation analysis detected significant associations between group-specific taxa and several glycemic traits from T1 to T3, including oral glucose tolerance test (OGTT) 0-h glucose levels, OGTT 2-h glucose levels, FBG levels, and GA levels, all of which were positively correlated with hyperglycemia (Fig. 3). In brief, the PGDM-enriched taxa were generally correlated with higher glucose levels during pregnancy. These taxa included OTU23 (*Bacteroides*), OTU27 (*Bacteroides*), OTU80 (*Bacteroides*), and OTU88 (Roseburia intestinalis), which were positively correlated with at least one of the glycemic traits mentioned above. OTU16 (*Megamonas*), however, was correlated with lower OGTT 2-h glucose levels. Conversely, nine taxonomic biomarkers that were consistently enriched in the women with normal glucose levels, namely, OTU1 (Bacteroides vulgatus), OTU3 (Faecalibacterium prausnitzii), OTU4 (Eubacterium rectale), OTU30 (Streptococcus oralis), OTU31 (Prevotella melaninogenica), OTU33 (Neisseria perflava), OTU35 (Bacteroides ovatus), OTU43 (Veillonella dispar), and OTU46 (Haemophilus parainfluenzae), exhibited negative positive correlations with at least one of the glycemic traits mentioned above in all trimesters.

### Patterns in bacterial interactions in PGDM and normoglycemic pregnancies.

**FIG 4:**
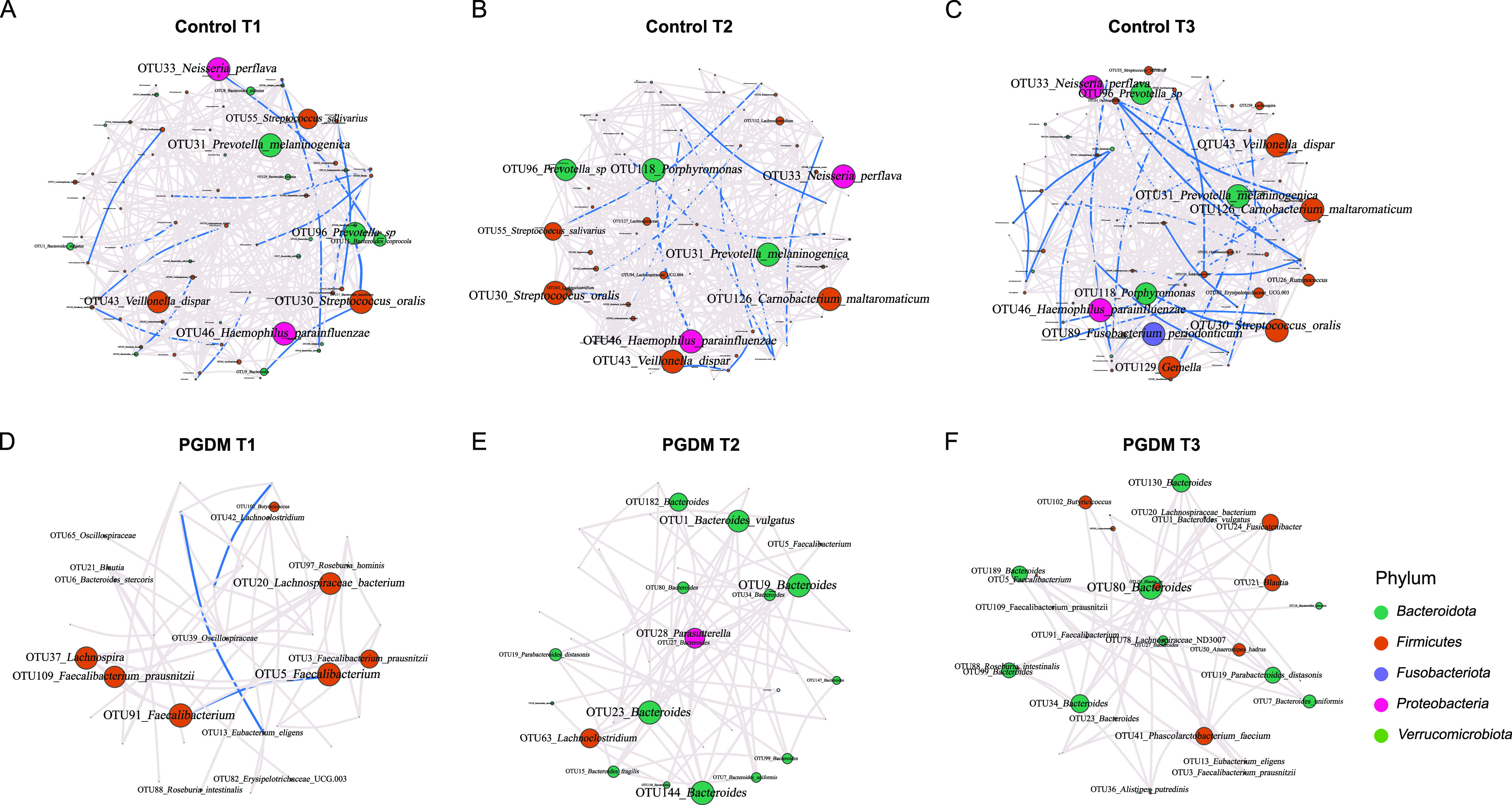
Gut microbial networks in pregnant women with or without PGDM in different trimesters. (A to C) Control group from T1 to T3. (D to E) PGDM group from T1 to T3. Nodes represent OTUs; the sizes indicate the eigenvector centrality, and the colors indicate the phylum of each OTU. Edges represent microbial correlations; gray and blue indicate positive and negative correlations, respectively. Edge thickness indicates correlation strength, and only the high-confidence interactions (*Q *< 0.05) with high absolute correlation coefficients (>0.3) are presented.

We further constructed concurrence networks to investigate the patterns in gut microbiota interactions in the women with PGDM and healthy controls. The bacterial interactions exhibited relatively stable dynamics within each group from T1 to T3, and most were positively correlated with each other (Fig. 4). However, we found distinct differences in the microbial correlation networks between the two groups. The hyperglycemic pregnancies exhibited fewer nodes and edges and lower average degrees (the average number of connections per node) but higher modularity (indicator of how well a network decomposes into modular communities) than the control group, indicating that the overall bacterial interactions in the women with PGDM were weaker than those in the women with normoglycemia. Of note, in the 10 most connected OTUs in the PGDM group, the proportion of taxa belonging to *Bacteroidetes* increased from T1 to T3, whereas that belonging to *Firmicutes* decreased in comparison with the control group (Table S6).

## DISCUSSION

To our knowledge, this study is the first to demonstrate that pregnant women with PGDM have altered gut microbial compositions compared with women who have healthy pregnancies. Differences in taxon composition and microbial networks between the women with PGDM and healthy controls were identified from T1 to T3. Furthermore, the taxonomic shifts in the gut bacteria distinctly decreased in the women with hyperglycemia as their pregnancies progressed. These findings contribute to the understanding of the association between the gut microbiota and the clinical outcomes of T2DM during pregnancy.

Our data revealed profound remodeling of the gut microbiota in the participants with normoglycemia during pregnancy. In the healthy controls, the alpha and beta diversities decreased, the *Firmicutes*/*Bacteroidetes* ratio increased, and the relative abundances of specific OTUs associated with insulin resistance and T2DM, such as *Bacteroides* and *Blautia* sp., increased as the pregnancies progressed. Moreover, one OTU, namely, Roseburia inulinivorans, was enriched in the healthy individuals, and its abundance significantly decreased from T1 to T3 (11, 16, 21). These taxonomic changes were consistent with those reported in previous studies on the changes in the gut microbiota during pregnancy (15–19).

The pregnant women with hyperglycemia had a lower richness in the different trimesters and fewer changes in alpha diversity with gestational age than the women with healthy pregnancies. The hyperglycemic women also showed an opposite trend in beta diversity compared to the healthy controls. In the PGDM group, the intertrimester variability of the gut microbiota decreased markedly. The lack of broad dynamic changes in microbial diversity in the women with PGDM suggested that such changes may be independent of the effects of pregnancy. Moreover, *Megamonas*, a genus of *Bacteroidetes*, was consistently enriched in the PGDM group during pregnancy. The abundance of *Megamonas* has been found to be positively correlated with FBG and GA levels in patients with T2DM (22, 23). It is also associated with obesity and inflammation (24–26), suggesting that it may play a role in T2DM pathophysiology. However, we detected a negative correlation between *Megamonas* and OGTT 2-h glucose levels, which was contradictory to the findings in previous studies. Therefore, further studies are needed to determine the role of *Megamonas* in the metabolic status of T2DM during pregnancy. In addition, bacteria such as Faecalibacterium prausnitzii, Roseburia inulinivorans, *Subdoligranulum*, Veillonella dispar, *Christensenellaceae*, *Lachnospiraceae*, and Prevotella melaninogenica were significantly depleted in the women with PGDM. This observation was consistent with that in patients with T2DM compared with healthy individuals (11, 21, 27–29). Most of these bacteria can produce short-chain fatty acids, particularly butyrate, which can improve insulin sensitivity by maintaining normal intestinal functions, regulating gut permeability, increasing gut hormone secretion, and suppressing proinflammatory cytokine production (11, 21, 30, 31).

We also noticed a distinct difference in the gut microbial networks between the women with and without hyperglycemia. In general, most of the gut bacteria were positively correlated with each other in both groups. However, the PGDM group exhibited weaker microbial interactions in comparison with the healthy controls. Moreover, the women with PGDM had a greater abundance of *Bacteroidetes*, while the proportions of *Firmicutes*, particularly those of butyrate-producing bacteria, decreased. These opposite interaction patterns between the two groups suggest that normoglycemic individuals may have a positive feedback mechanism that can maintain normal gut function by generating a sufficient amount of butyrate, while pregnancies affected by PGDM lack these beneficial microbial networks but have more intense positive interactions involving *Bacteroidetes*, which may be related to the reduced production of butyrate and consequent increased risk of low-grade inflammation and gut permeability. Further studies are needed to determine whether gut microbial networks are related to PGDM metabolic status and pregnancy outcome.

Our study enhances the understanding of the correlations between specific OTUs and maternal clinical indices. As expected, the OTUs that were overrepresented in the PGDM group were more likely to be correlated with abnormal glycemic traits. In addition, some of the genera showed divergent correlations with the same indices between the two groups, suggesting that different strains within a genus may be involved. Shotgun-based sequencing studies on the gut microbiome in PGDM may be performed in the future to better elucidate these findings.

Overall, we investigated the dynamics of the gut microbiota during pregnancy in women with PGDM and healthy controls and identified the taxa with differential abundances in different trimesters, which may be correlated with maternal metabolic status during pregnancy. However, this study was limited by the sample size of the case group and the lack of fecal samples before pregnancy. Given that this was an observational study, we could not exclude the existence of certain confounding factors. For instance, 20% of the women with PGDM in our study used oral metformin combined with insulin injections as their antidiabetic therapies. Metformin markedly affects the intestinal microbiota composition (32); however, because of the small size of the PGDM group, we could not correct the potential confounding factor of metformin during the assessment of the gut microbiome of the patients with PGDM.

In summary, this study reveals the distinct characteristics of the gut microbiota composition with gestational ages between normoglycemic and PGDM-affected pregnancies, including the altered gut microbiota composition, reduced intertrimester variability, certain associations between specific taxa abundances, and changes in gut microbial networks. Our findings indicate that interventions modulating the gut microbiota may be promising strategies for the clinical management of pregnant women with hyperglycemia. Further longitudinal studies involving women with T2DM at preconception stages and investigations using shotgun metagenomic sequencing should be performed to elucidate the relationships between specific bacterial species and PGDM metabolic statuses during pregnancy and thereby identify potential therapeutic targets for these conditions.

## MATERIALS AND METHODS

### Patient recruitment and sample collection.

This study was approved by the institutional review board of the Peking Union Medical College Hospital, Chinese Academy of Medical Science (date of approval, 27 March 2018; reference number JS-1535). Written informed consent was obtained from all of the eligible participants in accordance with the principles of the Declaration of Helsinki. This study was organized and reported in accordance with the principles of the STORMS reporting guidelines.

Twenty patients with PGDM were consecutively enrolled in Peking Union Medical College Hospital from April 2018 to December 2020. Pregnant women ≥18 years old were recruited during their first trimester (7 to 14 weeks) if diabetes had been diagnosed prior to pregnancy with the standard diagnostic criteria of a hemoglobin A1C (HbA1C) value of ≥6.5%, fasting plasma glucose of ≥7.0 mmol/L, or 2-h glucose of ≥11.0 mmol/L in a 75-g OGTT. Exclusion criteria were as follows: antibiotic treatment, proton pump inhibitors in the last 3 months, prebiotic or probiotic intake in the last 3 months, and presence of intestinal bowel disease. Control participants with normal blood glucose levels throughout their pregnancies were recruited and matched to the confirmed PGDM cases at a ratio of 1:3 with respect to age (±3 years), gestational age (±1 week), and sample collection date (±1 month). The 75-g OGTT results for all of the controls were in compliance with the standards proposed by the American College of Obstetricians and Gynecologists for gestational diabetes mellitus, which require fasting, 1-h, and 2-h blood glucose values to be lower than 5.1, 10.0, and 8.5 mmol/L, respectively (33, 34).

### Clinical data collection.

The following data were recorded for each participant: age, family history, diagnostic duration of T2DM, complications, gravidity, parity, method of conception, expected date of confinement, antibiotic treatments, height, weight at the first visit, preconception weight, weight gain during pregnancy, blood pressure, delivery method, and neonatal outcome (gender, birth weight, and NICU admission).

Laboratory data were extracted from electronic medical records, including 75-g OGTT results, fasting insulin levels, GA, HbA1C, fasting venous blood glucose, postprandial venous blood glucose, C-reactive protein, alanine aminotransferase, serum creatinine, and serum lipid profiles.

### Sample collection.

Stool samples from the participants with PGDM and healthy controls were collected during T1, T2, and T3. The samples were delivered to the laboratory within 3 h after collection and stored at −80°C until further processing.

### DNA extraction and 16S rRNA gene amplicon sequencing.

Total genomic DNA was extracted from the fecal samples (200 mg) using the QIAamp Fast DNA stool minikit (Qiagen, Hilden, Germany) per the manufacturer’s instructions. The hypervariable V4 region of the bacterial 16S rRNA gene was amplified using specific 515F and 806R primers with their barcodes (35). PCR was performed using the 2× Kapa library amplification ReadyMix (Roche, Boston, MA, USA). The PCR products were purified using the QIAquick gel extraction kit (Qiagen, Hilden, Germany) following the manufacturer’s instructions and quantified using Qubit 4.0 (Invitrogen, Carlsbad, CA, USA). Ultrapure water was used as a negative control during the extraction and PCR procedures to detect potential contamination. A sequencing library was constructed using the TruSeq DNA PCR-Free sample preparation kit (Illumina, Heyward, CA, USA) per the manufacturer’s instructions and sequenced on the Illumina HiSeq 2500 platform to generate 250-bp paired-end reads.

### Processing of the 16S rRNA sequencing data.

Raw paired-end reads were obtained using FLASH (version 1.2.11) and filtered with mothur (version 1.31.2). High-quality sequences with ≥97% similarity were assigned to the same OTUs using USEARCH (version 7.0.1090). Chimeras were filtered against the GOLD database (v20110518) using UCHIME (v4.2.40). The seed sequences for each OTU were annotated using taxonomic information from the SILVA database (V138; https://www.arb-silva.de) with the RDP classifier (version 2.2).

### Statistical analysis.

The data were analyzed using R version 4.0.2 and SPSS version 20.0 (SPSS Inc., Chicago, IL, USA). For the clinical data analysis, quantitative data were reported as means and standard deviations or medians and interquartile ranges, where appropriate. Normally distributed quantitative data were examined using Student’s *t* tests; otherwise, the Wilcoxon rank sum test (also called the Mann–Whitney U test) was performed. Qualitative data are presented as numbers (percentages) and were evaluated using the χ^2^ test or Fisher’s exact probability test. Data with *P* values of <0.05 were considered statistically significant.

Alpha diversity indices, including Chao1, Shannon-Wiener, and Simpson’s indices, were calculated based on the OTU levels (R vegan 2.5-7). The Friedman test was performed to compare the data from different trimesters, and the Wilcoxon rank sum test was conducted to compare the data from the PGDM and case groups, including OTU/phylum relative abundances and alpha diversity indices. The Bray-Curtis dissimilarities of the relative abundances of the OTUs between the groups or among the trimesters were calculated using R vegan. PERMANOVA was used to assess statistical significance based on the Bray-Curtis dissimilarity. The *Firmicutes*/*Bacteroidetes* ratios between two groups were evaluated using Welch’s *t* test.

Significant OTU features between the PGDM and case groups were identified using a linear discriminant analysis (LDA) effect size with an LDA score above 2. Significant OTU features between the PGDM and case groups adjusted for confounding variables were identified using LASSO regression: (i) we chose features that occurred in at least 10% of the samples and had a mean abundance of at least 0.01% and then centered and scaled the OTU data; (ii) we performed 10-fold cross-validation and identified lambda.1se within 1 standard error of the minimum; (iii) we fitted the LASSO regression model and considered unbalanced variables, such as preconception BMI and PCOS, using the glmnet (v4.1-3) function of the R package. Significant features between the PGDM and case groups and significant OTU features among the different trimesters were identified using the Friedman test based on features that occurred in at least 10% of the samples and had a mean abundance of at least 0.01%. *P* values were corrected for multiple testing using the Benjamini–Hochberg method, and data with false discovery rates (FDRs) of <0.05 were considered statistically significant.

Spearman’s correlation coefficients were calculated to investigate associations between the OTUs and clinical indices in the different trimesters. The package pheatmap in R was used to visualize the results.

Associations between the OTUs were assessed using Spearman’s correlation coefficients with Benjamini-Hochberg FDR corrections for multiple testing (significance threshold [*Q*] < 0.05). All of the included OTUs were detected based on a relative abundance of at least 0.001 in >50% of the samples in each group. Significant correlations with high absolute correlation coefficients >0.3 were visualized using the R package igraph (v1.2.11). The characteristics of the microbial correlation networks were also calculated using igraph (v1.2.11).

### Data availability.

The sequencing data that support the findings of this study are available in the CNGB Nucleotide Sequence Archive (CNSA [https://db.cngb.org/cnsa]; accession link, https://db.cngb.org/cnsa/project/CNP0003237_5339c6e4/reviewlink/). The clinical data for the participants and the STORMS checklist are available in the Mendeley Data Repository (https://data.mendeley.com/datasets/jjjd7n8gyp/1).

10.1128/msystems.01146-22.8 TABLE S6 Characteristics of gut microbial networks in pregnant women with or without PGDM in different trimesters. Download Table S6, XLSX file, 0.01 MB. Copyright © 2023 Ren et al. 2023 Ren et al. https://creativecommons.org/licenses/by/4.0/ This content is distributed under the terms of the Creative Commons Attribution 4.0 International license.
